# The Roles of Patients’ Authenticity and Accepting External Influence, and Clinicians’ Treatment Styles in Predicting Patients’ Dental Anxiety and Avoidance of Dental Appointments

**DOI:** 10.5964/ejop.v16i1.1664

**Published:** 2020-03-03

**Authors:** Anne Elisabeth Münster Halvari, Hallgeir Halvari, Edward L. Deci

**Affiliations:** a Faculty of Dentistry, Department of Dental Hygienist Education, University of Oslo, Oslo, Norway; b Department of Health, Social and Welfare Studies, University of South-Eastern Norway, Bakkenteigen, Norway; c Department of Business, Marketing and Law, University of South-Eastern Norway, Hønefoss, Norway; d Department of Clinical and Social Sciences in Psychology, University of Rochester, Rochester, NY, USA; e Institute for Positive Psychology and Education, Australian Catholic University, Strathfield, NSW, Australia; Edinburgh Napier University, United Kingdom

**Keywords:** authentic living, accepting external influence, autonomy support, controlling treatment style, dental anxiety, avoiding dental appointments

## Abstract

A substantial proportion of adults suffer from high dental anxiety, which is related to poor oral health and functioning. Using authenticity theory and self-determination theory, we applied a model testing two moderated mediation hypotheses: (i) the negative indirect association between authenticity and avoiding dental appointments through dental anxiety would be more evident when clinicians provides higher levels of autonomy support; and (ii) the indirect positive association between accepting external influence and avoiding dental appointments through dental anxiety would be more evident when clinicians provides higher levels of controllingness. Participants (N = 208) responded to a survey with validated questionnaires. The model with hypotheses were tested using Structural Equation Modeling (SEM) in LISREL and Conditional Process Modeling (moderated mediation). The results supported our hypotheses. The SEM model tested was found to fit the data well. Patient’s personality and dental clinic treatment environments predicted 38% of the variance in dental anxiety, which explained 38% of avoidance of treatment.

Dental anxiety is defined as fear of dental treatment or certain aspects of it (ter Horst & de Wit, 1993). A substantial proportion of adults suffer from high dental anxiety (Johnsen et al., 2003), which is related to avoiding dental appointments, and poor oral health and functioning (Halvari, Halvari, & Deci, 2018). Research on dental anxiety have addressed both patients’ previous distressing dental experiences and personality. Distressing dental experiences positively linked to patients’ dental anxiety have been oral-health-care clinicians’ treatment styles perceived as non-understanding or controlling. In addition, patients’ feeling extreme helplessness and embarrassment during dental treatments, the pain of having a tooth drilled, and the receipt of injections have been positively correlated with dental anxiety (Humphris & King, 2011; Halvari, Halvari, Bjørnebekk, & Deci, 2010; Oosterink, de Jongh, & Aartman, 2009). Regarding personalities, dental anxiety has been positively associated with neuroticism (Vassend, Røysamb, & Nielsen, 2011), general and phobic anxiety, and depression (Eli, Uziel, Baht, & Kleinhauz, 1997; Pohjola, Mattila, Joukamaa, & Lahti, 2011). Besides this, dental anxiety has been positively correlated with negative emotionality and fearfulness (Arnrup, Broberg, Berggren, & Bodin, 2007), as well as negatively with cooperativeness (Bergdahl & Bergdahl, 2003). Further, dental anxiety has been positively associated with external locus of control or the feelings of being pressured by others or environmental forces to behave (Poulton, Waldie, Thomson, & Locker, 2001). Hence, previous research has been largely associated with personality constructs that increase dental anxiety. Therefore, the present study focused on the authentic personality, which is expected to increase wellness such as reductions in general anxiety and negative affect, and increases in well-being (Gillath, Sesko, Shaver, & Chun, 2010; Sheldon, Ryan, Rawsthorne, & Ilardi, 1997). In addition, the current study also focused on the personality trait of accepting external influence, which has been associated with higher general anxiety and lower well-being (Wood, Linley, Maltby, Baliousis, & Joseph, 2008). Consequently, our research questions proposed are: What relations are there between patients authentic and accepting-external-influence personalities and dental anxiety? Are these personalities indirectly associated with avoiding dental appointments through dental anxiety? Does oral-health-care clinicians’ treatment styles moderate these indirect associations?

## The Authentic and Accepting-External-Influence Personalities

Authenticity and accepting external influence are defined according to Rogers (1959, 1961) person-centered theory of personality. First, authenticity or authentic living involves being true to oneself in most situations (Wood et al., 2008), which means living in accordance with one’s emotions, values, goals, and beliefs. Second, the term accepting external influence from other people is described as the belief that one has to conform to social influences such as peer pressure, expectations of others, and social norms (Gillath et al., 2010; Wood et al., 2008).

**Figure 1:**
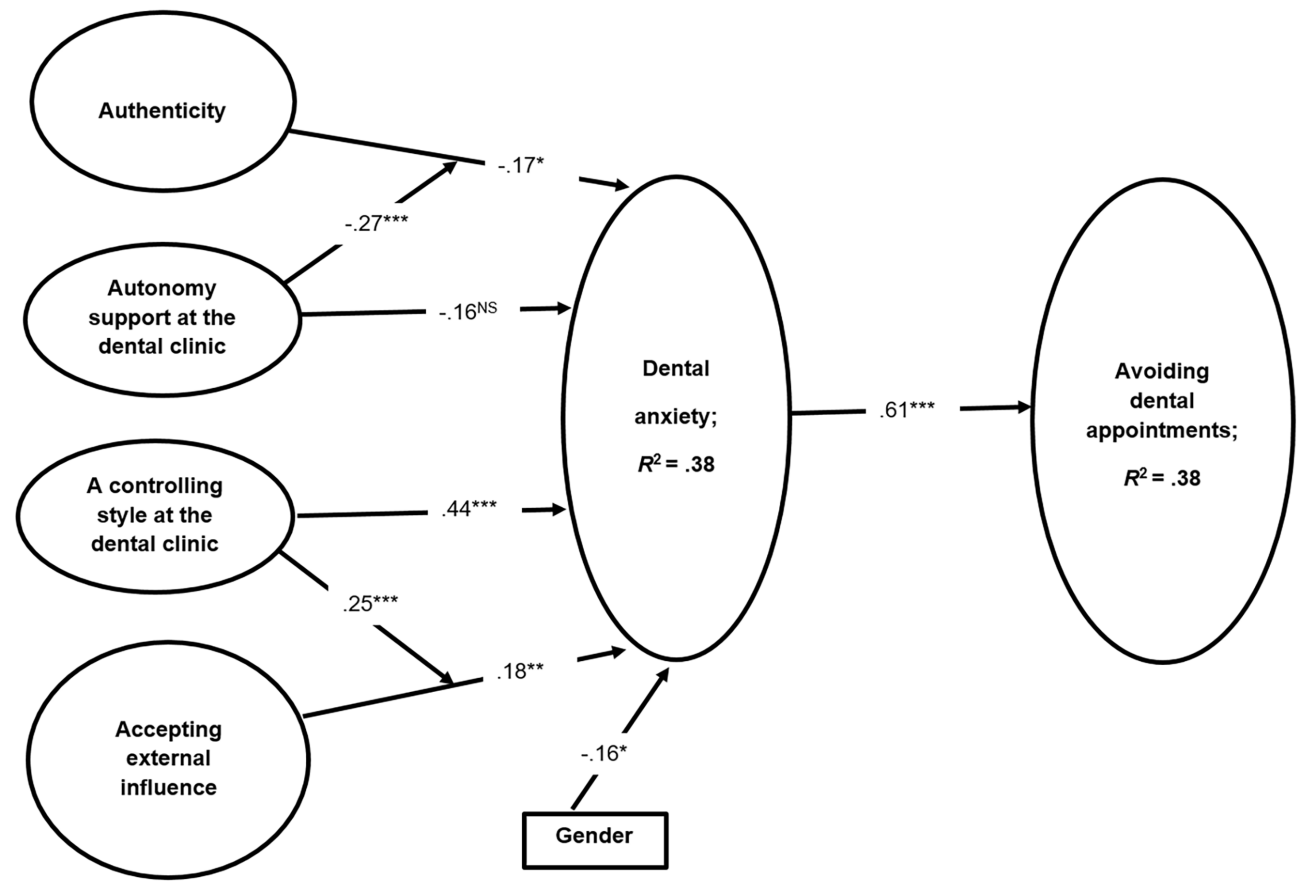
Standardized parameter estimates in the latent structural equation model of dental anxiety and avoidance of dental appointments. Due to presentation clarity, all factor loadings are presented in the method section. *p < .05. **p < .01. ***p < .001. NS = Not Significant.

Psychologists view authenticity as a precursor to well-being and healthy functioning (Horney, 1951; May, 1981; Rogers, 1961). Hence, as anxiety is conceived as a negative aspect of psychological well-being, the authentic personality might be expected to mitigate anxiety (i.e., to increase well-being). Research among people adopting other social roles than the role of a patient (i.e., mother, father, student, roommate, employee, teacher, child, friend, romantic partner) have shown that greater authenticity is associated with higher well-being, less neuroticism and more extraversion, openness, and agreeableness in samples representing people in different countries (Lynch, LaGuardia, & Ryan, 2009; Sheldon, Ryan, Rawsthorne, & Ilardi, 1997). Another study showed that the trait of accepting external influence was significantly positively linked to anxiety (Wood et al., 2008), and in a study by Gillath and colleagues (2010) trait authenticity was negatively related to attachment anxiety and avoidance. Consequently, based on the above evaluation of studies, both the authentic and external oriented personalities, respectively, seem to be substantially related to anxiety. In the model illustrated in Figure 1, the authenticity and accepting external influence constructs, conceived as personality variables, are located to the left, together with the constructs of perceived autonomy supportive and controlling treatment styles, which affects dental anxiety, and subsequently avoidance of dental appointments. Thus, according to the model and the research described above, the authentic personality is expected to be negatively associated with dental anxiety, whereas accepting external influence is expected to be positively related to dental anxiety.

## Self-Determination Theory: Autonomy-Supportive and Controlling Treatment Styles

Oral-health-care clinicians’ treatment styles may contribute to the interpretation of dental anxiety. An autonomy-supportive treatment style is defined by an oral health care professional who offer choice, provide a meaningful rationale, minimize pressure, and acknowledge the patients’ feelings and perspectives (Williams, Grow, Freedman, Ryan, & Deci, 1996). Conversely, a controlling oral-health-care clinician style is associated with pressuring and coercing oral communication, and evaluation making the patient belittled, humiliated, and embarrassed (Halvari et al., 2010). Research based on Self-Determination Theory (Deci & Ryan, 2000) indicated that authenticity is greater when autonomy support is experienced and is undermined by the experience of controlling environments (Lynch & Sheldon, 2017; Ryan & Ryan, 2018).

Research on the relations between oral-health-care clinicians’ autonomy supporting and controlling treatment styles, and dental anxiety, indicate that autonomy support is negatively correlated with dental anxiety, and a controlling style is positively correlated with dental anxiety (Halvari et al., 2010; Halvari et al., 2018). Multivariate analyses of the data from these two studies indicate that oral-health-care clinicians’ controlling styles are a stronger predictor of dental anxiety than autonomy support, but their moderating mechanisms of possible links between personality, dental anxiety, and avoidance of dental appointments has never been investigated. Research does also indicate that dental anxiety and avoiding dental appointments are strongly positively correlated (Halvari et al., 2010; Halvari et al., 2018).

## The Moderated and Moderated Mediation Roles of Treatment Styles

Treatment styles were expected to moderate the links between personality and dental anxiety. A study indicated that an autonomy-supportive intervention was more effective in increasing dental attendance among highly autonomously oriented personalities than among those low on this construct (Halvari, Halvari, Williams, & Deci, 2017). These results are in line with a meta-analysis, although not including studies on dental care, of the effectiveness of intervention programs designed to support autonomy, which resulted in greater effect sizes among autonomous (rather than control) oriented participants (Su & Reeve, 2011). Authentic behavior is per definition autonomous, as autonomy mean that one is acting in ways congruent with one’s values, preferences, and needs (Kernis & Goldman, 2006). Hence, because the authentic and the autonomous personalities share important characteristics, and seem to function accordingly, autonomy support seems likely to moderate the link between authenticity and dental anxiety, such that autonomy support would show greater mitigation of dental anxiety and increase health-related behavior (i.e., dental attendance) among patients who are high in authenticity (in relation to those low in authenticity). Conversely, a study by Grolnick and Ryan (1987) indicate that experimentally induced learning conditions that are controlling (in relation to less controlling conditions) resulted in more pressure and anxiety and less learning, presumably because they were more conducive to controlled motivation or an external locus of causality, that is, a construct similar to accepting external influence as used in the present study. Moreover, anxiety seem to be reduced more among persons high in accepting external influence (relative to those low in accepting external influence) if they experience decreased need frustration (i.e., indication of lower levels of controllingness) (Bryan, Baker, & Tou, 2017).

## Possible Control / Background Variables

Gender was included in the study as a possible control variable because women have universally reported more dental anxiety than men (Hill et al., 2013; Liddell & Locker, 1997; ter Horst & de Wit, 1993). In addition, we included age and some socioeconomic factors as background variables because they are shown to be important for population access to the health services and health (e.g., Sreebny, 1983). However, among students who pursue important educational life aspirations at the university, socioeconomic factors tend to be less important than in other sub-populations (Halvari, Halvari, Bjørnebekk, & Deci, 2012a). On the other hand, if they are important we have the possibility to control for them in the model testing.

## Hypotheses

The following hypotheses (H) were specified based on the literature reviewed: (H1) authenticity will be negatively associated with dental anxiety; (H2) autonomy support will be negatively associated with dental anxiety; (H3) accepting external influence will be positively associated with dental anxiety; (H4) a controlling treatment style will be positively associated with dental anxiety; and (H5) dental anxiety will be positively associated with avoiding dental appointments. Further, the following indirect associations were tested: (H6) there will be a negative indirect relation between authenticity and avoiding dental appointments through dental anxiety; and (H7) there will be a negative indirect relation between autonomy support and avoiding dental appointments through dental anxiety. Next, (H8) there will be a positive indirect relation between accepting external influence and avoiding dental appointments through dental anxiety; and (H9) there will be a positive indirect relation between a controlling treatment style and avoiding dental appointments through dental anxiety. Furthermore, (H10) autonomy-supportive treatment styles will moderate the association between authenticity and dental anxiety; (H11) controlling treatment styles will moderate the association between accepting external influence and dental anxiety; (H12) the indirect negative association between authenticity and avoiding dental appointments through dental anxiety will be more evident among those who experience higher levels of autonomy-supportive treatment styles; (H13), and the indirect positive association between accepting external influence and avoiding dental appointments through dental anxiety will be more evident among those who experience higher levels of controlling treatment styles. Lastly, (H14) women would report more dental anxiety than men.

## Method

### Participants

Norwegian student patients from 42 study disciplines at the University of Oslo were invited to participate in the study. They were informed about the aim of the study and gave their informed consent to participate. No incentives were offered for participation. A total of 520 questionnaires (physical copies) were handed out and 208 were returned (40%). Participants’ ages ranged from 18 to 45 years (*M* = 25.2, *SD =* 4.97). More females than males responded to the questionnaire (*n*_females_ = 146; 70.2%).

### Translation of Measures and Their Reliabilities

Following the procedures suggested by Beaton, Bombardier, Guillemin, and Ferraz (2000), all questionnaire measures described below, except perceived controllingness which is developed in Norway, were translated to Norwegian indepently by two researchers. Discrepancies in translations were resolved and agreed upon. Back-translation to English was performed by an expert in Norwegian and English language, and discrepancies between the original version and the back-translation were resolved, consensus reached among the expert and the two researchers, and the Norwegian versions adjusted when appropriate. Finally, the questionnaire items were pretested among 10 students in order to secure a valid understanding of item content meaning. Reliabilities of the scales are presented in Table 1.

### Design of Questionnaire

Before the patients responded to the items in the questionnaire of the present study they were introduced to their own clinic context by the following instructions and questions: “Think back to your last visit to a dental hygienist or dentist. It is important that you try to think about the treatment and your experiences with this oral-health-care clinician”. This introduction was followed by questions on who this oral health care professional was (a dental hygienist or a dentist, a female or a male), the number of visits to this oral health care professional, type of clinic (private or public), and time since last visit. “If you answered “dental hygienist” in question 1, please have this person in mind and answer the following questions with reference to your dental hygienist. However, if you answered “dentist” in question 1, please answer the following questions with reference to your dentist”. Of the respondents, 84% recalled their dentist.

### Questionnaires

#### Authenticity

Authenticity was measured with the Authenticity Scale developed by Wood and colleagues (2008). A sample item for authenticity (4 items) is “I live in accordance with my values and beliefs”; and for accepting external influence (4 items) it is “I am strongly influenced by the opinions of others”. Responses could vary from 1 (*does not describe me at all*) to 7 (*describes me very well*).

#### Perceived Autonomy Support

Perceived Autonomy Support was measured with the 6-item version of the modified Health-Care Climate Questionnaire (Williams et al., 1996), which was adapted to oral health care. A sample item is: “I feel that my oral-health-care clinician has provided me choices and options.” Responses could vary from 1 (*strongly disagree*) to 7 (*strongly agree*).

#### Perceived Controllingness

Perceived Controllingness at the Dental Clinic was measured with the 6-item Perceived Controlling Style at the Dental Clinic Questionnaire (PCSDCQ; Halvari et al., 2012a). A sample item is: “I find that my oral-health-care clinician decides too much”. Responses varied from 1 (*strongly disagree*) to 7 (*strongly agree*).

#### Anxiety for Dental Treatment

Anxiety for Dental Treatment was measured by the 5-item Modified Dental Anxiety Scale (Humphris et al., 2013). An example item is: “If you were sitting in the waiting room (waiting for treatment), how would you feel?” Participants responded on a 5-point scale ranging from 1 (*not anxious*) to 5 (*extremely anxious*).

#### Avoiding Dental Appointments

Avoiding dental appointments was measured by two questions: “Has fear or worry ever caused you to put off making an appointment 1) with a dental hygienist? 2) with a dentist?” Responses were: 1 (*never*), 2 (*once or twice*), 3 (*a few times*), 4 (*often*), and 5 (*nearly every time*). (Milgrom, Weinstein, & Getz, 1995).

#### Reliability

In previous research, *reliability coefficients* exceeded .70 for all scales, that is, for authenticity (Wood et al., 2008), autonomy support (Williams et al., 1996), controllingness (Halvari et al., 2010), anxiety (Humphris et al., 2013), and for avoiding dental appointments (Halvari et al., 2010). For reliability coefficients in the present study, see Table 1. They are all acceptable as they exceed the cut-point of .70 defined by Nunnally (1979).

#### Background Assessments

Gender (1 = *female* and 2 = *male*). Age was indicated in *years*. Educational level and socio-economic status information was taken care of by the following three questions: (1) “What is your highest completed education?” With response alternatives from 1 (*junior or senior high school*) to 5 (*university or university college education of more than 5 years*). (2) “How many *hours* per week do you work for income?” (3) “How would you describe your financial situation at the moment?” Response alternatives were from 1 (*very good*) to 5 (*very difficult*).

### Data Analysis

Correlational analyses were done using Pearson *r*, except for correlations involving gender in which Spearmans point bi-serial correlations were used.

The model illustrated in Figure 1, including the tests of indirect links, was tested simultaneously with the Structural Equation Model (SEM) using LISREL version 8.80. The two interaction terms (autonomy support X authenticity and controllingness X accepting external influence) were treated as observed variables in order to simplify the model. In addition, for the same reason and due to the large number of variables and indicators (i.e., scale items) relative to the sample size, two of them were parceled. That is, we randomly assigned items for autonomy supportive and controlling styles into 3 parcels, each with 2 items, as recommended by Little, Cunningham, Shahar, and Widaman (2002). All other variables in the model are latent in testing the model in Figure 1, factor loadings for items / parcels were all significant, and they were (loadings in parentheses) for authenticity (.84, .71, .54, .53), for accepting external influence (.82, .81, .72, .67), for perceived autonomy support (.90, .85, .82), for perceived controllingness (.84, .80, .73), for dental anxiety (.91, .82, .82, .77, .73), and for avoiding dental appointments (.91, .91).

To evaluate the fit of the model tested, we used the chi-square likelihood ratio (χ^2^), the Root Mean Square Error of Approximation (RMSEA), the Comparative Fit Index (CFI), and the Incremental Fit Index (IFI) as recommended (Bollen, 1989; Hu & Bentler, 1999). A good fit should have a value close to or lower than .06 for the RMSEA, a value close to or lower than .08 for the SRMR, and a value close to or higher than .95 for the CFI and IFI.

Regarding mediation, an indirect link is significantly supported if the bias-corrected 95% confidence intervals (for the bands of products of coefficients after 5000 resamplings) do not include zero or oppositely valued coefficients (see Table 2) (Preacher & Hayes, 2008).

The bootstrapping procedure described in Preacher, Rucker, and Hayes (2007) were used to examine moderation. These moderator relations were illustrated following the formula proposed by West, Aikin, and Krull (1996, pp. 35 – 36). Finally, moderated mediation was tested using Model 7 in Model Templates for PROCESS for SPSS and SAS (2013-2015) by Andrew F. Hayes (Preacher, Rucker, & Hayes, 2007).

## Results

### Descriptive Statistics and Reliability

The means, standard deviations, skewness values, and reliabilities for all variables emerge in Table 1. Reliability values are acceptable. Skewness values (skew < 3.0) are acceptable for use in SEM analysis (Kline, 2005). Regarding means and distribution of scores, for example, dental anxiety had a mean score of 11.05 in the current sample (2.21 pr. item X 5 items, see Table 1). This mean value for dental anxiety is almost the same as the mean value presented by Humphris et al. (2013) in their validation study of the Modified Dental Anxiety Scale. Of the participants, 54.8% had low scores (5-10; “not anxious”), 27.4% had moderate scores (11-15; “quite anxious”), and 17.8% had high scores (16-25; “very and extremely anxious”). Those with extreme dental anxiety (≥ 19%) were somewhat lower in the present sample (10.1%) compared to the Humphris et al. (2013) study (12.1), and the reliability coefficients (α) were .90 and .92, respectively. Regarding the perceived controlling treatment style, 54% responded with low scores (7-12; “disagree”), 35% with moderate scores (13-24; “somewhat agree”), and 11% responded with high scores (25-36; “agree”). The use of the above categories of low, moderate, high, and extreme dental anxiety scores were used to compare them with scores from the Humphris et al. (2013) validation article. In addition, the categories for low, moderate, and high scores for controllingness were given. For all other analysis described below all variable raw scores were used for all variables, including interactions illustrated using high and low scores (see West et al., 1996)

### Hypotheses Testing

We used SEM in testing the model with the direct, indirect, and moderated associations. In addition, we used the non-parametric bootstrapping procedure in testing moderated and moderated mediational associations (conditional indirect effects) (Preacher et al., 2007).

### Theoretical Model

The zero order correlations in Table 1 are all in line with the expectations. Education and socioeconomic variables were not significantly correlated with authenticity, accepting external influence, treatment styles, dental anxiety, or avoiding dental appointments.

### Empirical Models

The measurement model for Figure 1 was tested with all variables and indicators and found to fit the data well, χ^2^(178, *N* = 208) = 308.63, *p* < .001; RMSEA = .060, 95% CI [.048, .071]; CFI = .96; IFI = .96; SRMR = .059. The structural model (Figure 1) was tested with this measurement model included. In this measurement model all factor loadings were significant (see the data analysis section above). A negative covariance between authenticity and accepting external influence was added as suggested by previous research (Wood et al., 2008). The structural model yielded the same good fit as the measurement model, χ^2^(178, *N* = 208) = 329.56, *p* < .001; RMSEA = .061, 95% CI [.051, .072]; CFI = .96; IFI = .96; SRMR = .060. The standardized parameter estimates are shown in Figure 1, supporting Hypotheses 1, 3, 4, 5, and 14.

**Table 1:** Zero-Order Correlations^a^ and Descriptive Statistics Among Variables

Variable	1.	2.	3.	4.	5.	6.	7.	8.	9.	10.	11.
1. Authenticity	--										
2. Accepting external influence	-.39***	--									
3. Autonomy supportive style at the clinic	.33***	-.28***	--								
4. Controlling style at the clinic	-.25***	.26***	-.67***	--							
5. Dental anxiety	-.20**	.28***	-.32***	.41***	--						
6. Avoiding dental appointments	-.05	.14*	-.22***	.34***	.57***	--					
7. Gender	-.06	-.04	-.01	-.05	-.16*	-.10	--				
8. Age	.02	.07	.02	.05	-.02	-.02	.05	--			
9. Work for income (hrs/week)	.01	.05	.08	-.08	-.11	-.07	-.14	.22**	--		
10. Problems with private economy	-.07	.02	-.09	.11	-.02	.08	-.06	.22**	-.14*	--	
11. Education (highest completed)	.01	.00	-.03	.08	-.02	.12	-.01	.56***	.21**	.10	--
*M*	5.53	3.18	5.04	2.23	2.21	1.46	1.29	25.2	8.51	2.69	3.79
*SD*	0.86	1.16	1.23	1.12	0.97	0.96	0.46	4.97	10.24	0.78	1.46
Skewness	-0.35	0.27	-0.59	0.99	0.95	2.37	0.89	-1.70	1.40	-0.02	-0.01
Reliability (α)	.71	.84	.90	.86	.90	.86	--	--	--	--	--

*Note.* *N* = 208. Gender: 1 = females; 2 = males.

^a^Pearson correlations, except Spearmans point bi-serial correlations involving gender.

*p < .05. **p < .01. ***p < .001.

### Mediation

Using LISREL, we tested the indirect links in Figure 1 simultaneously with testing the structural model. It was hypothesized negative indirect links between both authenticity (Hypothesis 6) and autonomy support (Hypothesis 7), respectively, and avoiding dental appointments through dental anxiety. In addition, it was predicted positive indirect links between both accepting external influence (Hypothesis 8) and a controlling clinic style (Hypothesis 9), respectively, and avoiding dental appointments through dental anxiety. These predictions were supported, except the indirect association between autonomy support and avoiding dental appointments through dental anxiety (Hypothesis 7) (see Table 2).

**Table 2:** Tests of Mediation (LISREL) and Moderated Mediation (Bootstrapping) of Links Emerging in Figure 1

Independent variable (IV)		Mediator (M)		Dependent variable (DV)	Effect	*SE*	Z(a*b path)	BC 95% CI	
								*LL*	*UL*
*Mediations*									
1. Authenticity (A)	→	Dental anxiety	→	Avoiding dental appointments	-0.08	0.05	-1.75*	-0.18	0.02
2. Autonomy support (AS)	→	Dental anxiety	→	Avoiding dental appointments	0.09	0.07	1.24	-0.05	0.23
3. A X AS	→	Dental anxiety	→	Avoiding dental appointments	-0.13	0.04	-3.18***	-0.21	-0.05
4. Accepting external influence (EX)	→	Dental anxiety	→	Avoiding dental appointments	0.10	0.04	2.24**	0.02	0.18
5. Controlling clinic style (CS)	→	Dental anxiety	→	Avoiding dental appointments	0.23	0.07	3.09***	0.09	0.37
6. EX X CS	→	Dental anxiety	→	Avoiding dental appointments	0.15	0.05	2.98***	0.05	0.25
*Moderated mediations (conditional indirect effects)*									
7. Authenticity	→	Dental anxiety	→	Avoiding dental appointments					
For autonomy support 1 *SD* below the mean					0.001	0.08		-0.16	0.14
For autonomy support at the mean					-0.087	0.05		-0.20	0.003
For autonomy support 1 *SD* above the mean					-0.29***	0.06		-0.29	-0.05
8. Accepting external influence	→	Dental anxiety	→	Avoiding dental appointments					
For a controlling clinic style 1 *SD* below the mean					-0.01	0.04		-0.10	0.07
For a controlling clinic style at the mean					0.05	0.03		-0.01	0.11
For a controlling clinic style 1 *SD* above the mean					0.18***	0.06		0.06	0.31

*Note.* BC = bias corrected; CI = confidence interval; 5000 bootstrap samples; a-path = IV → M; b-path = M → DV; *LL* = lower limit; *UL* = upper limit.

*p < .05. **p < .01. ***p < .001. One-tailed tests.

### Analyses of Moderation and Moderated Mediation

**Figure 2:**
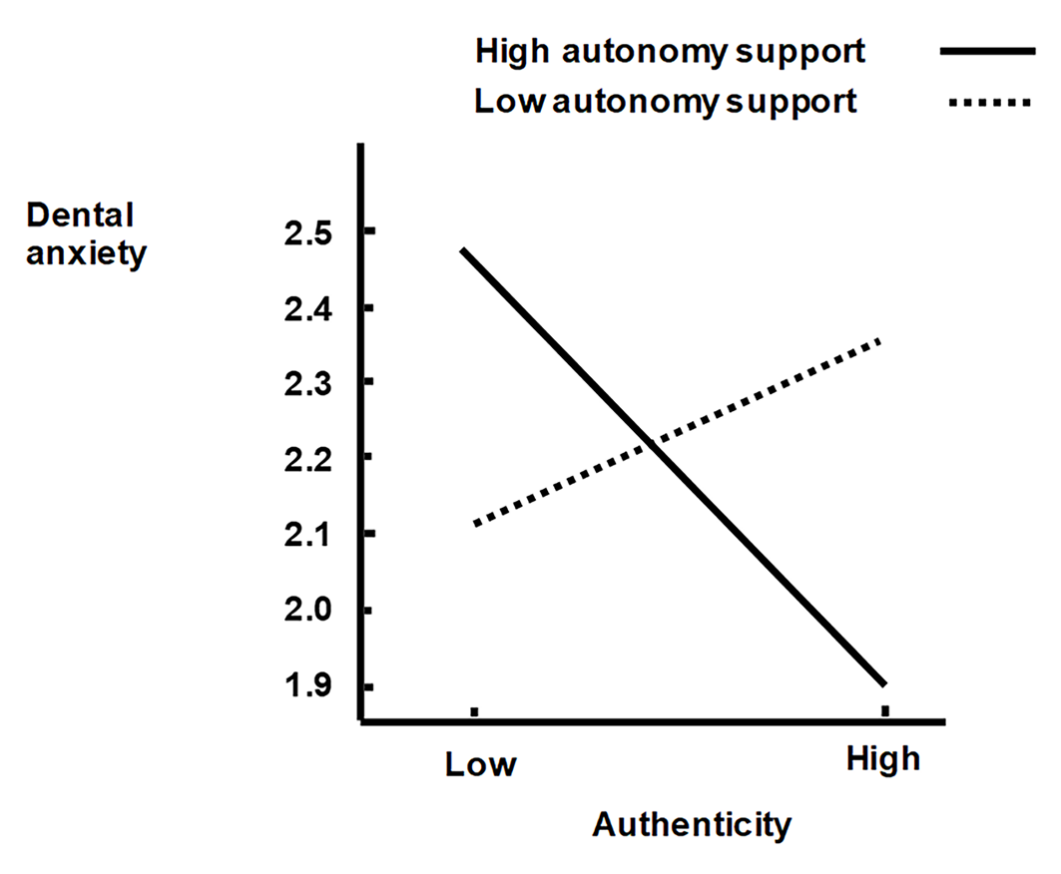
Simple regression lines depicting the relationship between authenticity and dental anxiety at specified values of autonomy support (Low = 1 *SD* below the mean whereas high = 1 *SD* above the mean).

**Figure 3:**
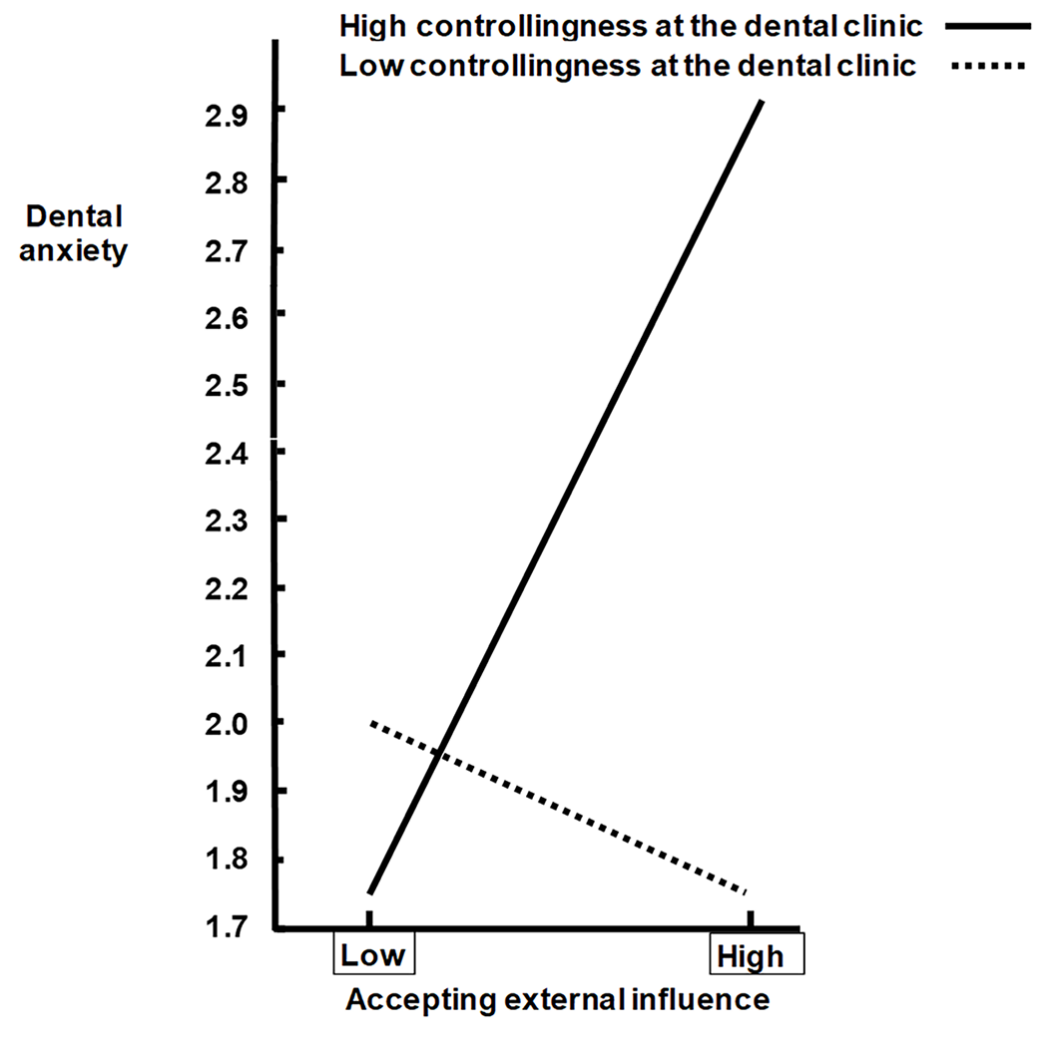
Simple regression lines depicting the relationship between an accepting external influence and dental anxiety at specified values of a controlling treatment style (Low = 1 *SD* below the mean whereas high = 1 *SD* above the mean).

For the moderation, the product of autonomy support and authenticity was hypothesized to be negatively associated with dental anxiety. This hypothesis 10 was supported (Coeff = -.11, *SE* = .05, *t* = -2.07, 95% BC CI [-.2220, -.0055] with 5000 resamples) *R*^2^_Change_ = .02, *F*(1, 201) = 4.30, *p* = .04, which means that the combination of high autonomy support and high authenticity would be associated with the lowest dental anxiety. In addition, the product of a controlling clinic style and accepting external influence was hypothesized to be positively associated with dental anxiety. This hypothesis 11 was also supported (Coeff = .14, *SE* = .04, *t* = 3.13, 95% BC CI [.0509, .2238] with 5000 resamples) *R*^2^_Change_ = .04, *F*(1, 201) = 9.81, *p* = .002, which means that the combination of a high controlling treatment style and high accept of external influence would be associated with the highest dental anxiety. These moderator relations were illustrated following the formula proposed by West, Aikin, and Krull (1996, pp. 35 – 36) (see Figures 2 and 3).

Regarding moderated mediations, we hypothesized that the negative indirect link between authenticity and avoiding appointments through dental anxiety would be more evident among those who experience higher autonomy-supportive treatment styles (Hypothesis 12). In addition, the positive indirect link between accepting external influence and avoiding dental appointments through dental anxiety would be more evident among those who experience higher controlling treatment styles (Hypothesis 11). These moderated mediation hypotheses were supported (see Table 2). The last Hypothesis 14 stating that women would be more dental anxious than men was also supported (see Table 1 and Figure 1).

## Discussion

The structural model of dental anxiety and avoiding dental appointments was tested and received good fit. Authenticity was negatively related to dental anxiety. Autonomy support did not directly predict anxiety, but moderated the authenticity – dental anxiety association, such that high authenticity is associated with the lowest dental anxiety in particular among patients’ perceiving high clinician autonomy support. Both accepting external influence and a controlling treatment style were positively related to dental anxiety. In addition, a controlling treatment style did moderate the accepting external influence – dental anxiety link, such that high external influence is associated with the highest dental anxiety in particular among patients’ perceiving a high controlling oral-health-care clinician style. Further, the results revealed a negative indirect association between authenticity and avoiding dental appointments through dental anxiety, and a positive indirect association between accepting external influence and avoiding dental appointments through dental anxiety. These indirect links were moderated by autonomy supportive and controlling oral-health-care clinician styles, respectively, such that the indirect negative relation between authenticity and avoiding dental appointments through dental anxiety was more evident among those who reported higher levels of autonomy support, and that the indirect positive relation between accepting external influence and avoiding dental appointments through dental anxiety was more evident among those who reported higher levels of a controlling oral-health-care clinician style. The last hypothesis stating that women would be more dental anxious than men was also supported. Education and socioeconomic factors did not correlate significantly with the personality constructs, oral-health-care clinician styles, dental anxiety, or avoidance of dental appointments. These findings are supported by other research (Halvari et al., 2012a), indicating that they are of minor importance among university students in Norway.

This is the first study showing that: (i) the authentic and accepting external influence personality constructs are related to dental anxiety; (ii) autonomy support moderates the indirect association between authenticity and avoiding dental appointments through dental anxiety, and (iii) a controlling treatment style moderates the indirect association between accepting external influence and avoiding dental appointments through dental anxiety.

Authentic persons being true to oneself in most situations indicate that, when they are supported to make healthy choices in their lives, their value and goal systems would help them to do so and to behave accordingly. Their values and goals are congruent, integrated in their self with their behavior, and they feel harmonious and report low degrees of conflict (Deci & Ryan, 2000; Ryan & Ryan, 2018). Thus, because authenticity is supposed to develop among persons receiving autonomy support (Deci & Ryan, 2000; Ryan & Ryan, 2018), the authentic person is supposed to be oriented toward and select autonomy-supportive elements in the social context they are familiar with from their history of learning. The interaction between authenticity and clinician autonomy support indicate that patients high in authenticity report the lowest dental anxiety if they perceive receiving high autonomy support (in relation to low autonomy support) from their oral-health-care clinicians (see Figure 2).

This is as expected and in line with a meta-analysis (Su & Reeve, 2011) and an autonomy-supportive oral health care intervention (Halvari et al., 2017) indicating that autonomy-oriented personalities showed a greater training or intervention effect than did participants who were less autonomy-oriented. This interaction effect is supposed to be related to the authentic personality experiencing more congruence with autonomy support, and feel this type of support more appealing than less authentic persons, because it is integrated in their values and goals (Deci & Ryan, 2000). Conversely, patients low in authenticity reported higher dental anxiety if they perceived high autonomy support (relative to low autonomy support; see Figure 2) from their oral-health-care clinicians. If they do not have the authentic value- or goal-system, autonomy support from oral-health-care clinicians may feel unfamiliar to them and perceived as not providing structure, leaving them feeling abandoned and thus causing conflict and anxiety. In particular, offering options of choice might be experienced as anxiety provoking because they do not have the values and goals integrated that would help them to choose. When receiving autonomy support, patients low in authenticity might experience higher cognitive conflict and might not assimilate the autonomy-supportive information into their existing ways of thinking about what is motivating them for oral health care (Su & Reeve, 2011). Such autonomy-supportive information might relate to oral-health-care professionals’ verbal explanation intended to help the patient understand why a behavior have personal utility, acknowledgement of negative feelings as legitimate when making requests of the patient being in conflict with his or her personal inclinations, and providing information about options, encouragement of making choices and initiation of patient action. Less autonomy oriented individuals might also act in a more defensive way to information about autonomy support (Hodgins & Knee, 2002), which would further increase the level of conflict with the autonomy support. However, patients low in authenticity can learn to respond well to autonomy support (Williams et al., 1996).

A controlling treatment style predicted dental anxiety, which is in line with previous research (Halvari et al., 2010, 2018). In addition, a controlling treatment style moderated the association between accepting external influence and dental anxiety (see Figure 3). These results are in accordance with a central hypothesis in self-determination theory (Deci & Ryan, 2000), that an increase in autonomy support (need support) or a reduction in controllingness would influence self-determined integration of motivation, better performance, and well-being. The result indicating that a reduction in controllingness (Figure 3) mitigate dental anxiety in particular among patients high in accepting external influence is promising.

Even though the level of controlling treatment styles might be relatively low, a one-time incident of bad experience may have very negative consequences for patients. Consequently, we should bear in mind its’ larger impact on individuals than positive events (Baumeister, Bratslavsky, Finkenauer, & Vohs, 2001), and its robustness in predicting maladaptive developmental outcomes (Kins, Soenens, & Beyers, 2012; Soenens, Vansteenkiste, & Luyten, 2010).

The negative link between gender and dental anxiety indicated that women had higher levels of dental anxiety than men, which is consistent with the literature (Hill et al., 2013). Regarding education and socioeconomic status variables, two factors that are known to influence oral health in some samples (Carmichael, Rugg, French, & Cranage, 1980), no significant correlations appeared with the other study variables. This is in line with other research among student patients (Halvari et al., 2012a).

### Practical Implications

It is worth noting that autonomy support, or the reduction in controllingness which is an element of autonomy support, are involved in both personality interactions, with authenticity and with accepting external influence. Oral-health-care clinicians use of autonomy support and their reduction of controllingness might be very important in reducing dental anxiety and avoiding dental appointments among patients. Autonomy support can be learned (Williams & Deci, 1996). Examples from oral-health-care interventions that could be utilised in training of clinicians are exercises eliciting and reflecting patient perspectives, providing options and a rationale for change when a recommendation is made, supporting patient initiatives, minimizing pressure and a controlling language, and remaining non-judgmental. According to self-determination theory, such support is of vital importance in patient oral health promotion (Halvari & Halvari, 2006; Halvari, et al., 2012a; Halvari, Halvari, Bjørnebekk, & Deci, 2012b).

### Strengths and Limitations

Many strengths and limitations apply to the present study. *First*, self-reports were appropriate for authenticity, accepting external influence, perceived autonomy support, controlling style, and dental anxiety. However, observed measures of avoiding dental appointments would have strengthened the design. Construct validity of the self-report measures were demonstrated in SEM. *Second*, the current sample is a convenience sample from a specific population of student patients. The sample was not selected to be representative of all student patients, so caution must be taken when it comes to generalizing the results. However, the purpose of the present study was to test the links between variables derived from Self-Determination Theory, which is an universal theory, assuming that constructs such as authenticity and dental anxiety are more or less present in all individuals (Deci & Ryan, 2000). This means we tried to maximize the internal validity of the study, assuming that the relations between variables would be the same independent of sample variations. *Third*, the study has the limitations associated with being cross-sectional and the absence of a design allowing randomized control and longitudinal data implies that conclusions regarding causality cannot be inferred (Bollen, 1989). The analysis of the hypothesized model was performed in SEM with latent variables, which is a strength because error variances are accounted for. Still, the arrows between variables do not imply causality.
